# Mechanism of allosteric activation of TMEM16A/ANO1 channels by a commonly used chloride channel blocker

**DOI:** 10.1111/bph.13381

**Published:** 2016-01-18

**Authors:** Chau M Ta, Aiste Adomaviciene, Nils J G Rorsman, Hannah Garnett, Paolo Tammaro

**Affiliations:** ^1^ Department of PharmacologyUniversity of OxfordOxfordUK; ^2^ Faculty of Life SciencesThe University of ManchesterManchesterUK; ^3^ OXION Initiative in Ion Channels and DiseaseUniversity of OxfordOxfordUK

## Abstract

**Background and Purpose:**

Calcium‐activated chloride channels (CaCCs) play varied physiological roles and constitute potential therapeutic targets for conditions such as asthma and hypertension. *TMEM16A* encodes a CaCC. CaCC pharmacology is restricted to compounds with relatively low potency and poorly defined selectivity. Anthracene‐9‐carboxylic acid (A9C), an inhibitor of various chloride channel types, exhibits complex effects on native CaCCs and cloned TMEM16A channels providing both activation and inhibition. The mechanisms underlying these effects are not fully defined.

**Experimental Approach:**

Patch‐clamp electrophysiology in conjunction with concentration jump experiments was employed to define the mode of interaction of A9C with TMEM16A channels.

**Key Results:**

In the presence of high intracellular Ca^2+^, A9C inhibited TMEM16A currents in a voltage‐dependent manner by entering the channel from the outside. A9C activation, revealed in the presence of submaximal intracellular Ca^2+^ concentrations, was also voltage‐dependent. The electric distance of A9C inhibiting and activating binding site was ~0.6 in each case. Inhibition occurred according to an open‐channel block mechanism. Activation was due to a dramatic leftward shift in the steady‐state activation curve and slowed deactivation kinetics. Extracellular A9C competed with extracellular Cl^−^, suggesting that A9C binds deep in the channel's pore to exert both inhibiting and activating effects.

**Conclusions and Implications:**

A9C is an open TMEM16A channel blocker and gating modifier. These effects require A9C to bind to a region within the pore that is accessible from the extracellular side of the membrane. These data will aid the future drug design of compounds that selectively activate or inhibit TMEM16A channels.

**Abbreviations**
A9C: anthracene‐9‐carboxylic acid
CaCC: calcium‐activated chloride channel
*E*_*rev*_: reversal potential
*V*_*m*_: membrane potential

## Tables of Links

**Table:** 

**TARGETS**
CaCC (TMEM16A)
TMEM16B

**LIGANDS**
Anthracene‐9‐carboxylic acid (A9C)
DIDS
SITS
Tamoxifen

These Tables list key protein targets and ligands in this article which are hyperlinked to corresponding entries in http://www.guidetopharmacology.org, the common portal for data from the IUPHAR/BPS Guide to PHARMACOLOGY (Pawson *et al*., 2014) and are permanently archived in the Concise Guide to PHARMACOLOGY 2013/14 (Alexander *et al*., 2013).

## Introduction

Calcium‐activated chloride channels (CaCCs) are activated by rises in the free intracellular Ca^2+^ concentration ([Ca^2+^]_i_) and depolarization of the membrane potential (*V*_*m*_). Thus, they link Ca^2+^ signalling with the cell's electrical activity. Our understanding of the physiology and pharmacology of CaCCs has progressed less rapidly than those of other Cl^−^ channel families. This was mainly due to the limited knowledge of CaCC molecular structure and paucity of specific pharmacological modulators (Hartzell *et al*., 2005; Pedemonte and Galietta, 2014).

*TMEM16A* or *anoctamin1* (*ANO1*) was identified as a CaCC‐encoding genes in 2008 (Caputo *et al*., 2008; Schroeder *et al*., 2008; Yang *et al*., 2008). The *TMEM16*/*anoctamin* family is composed of 10 genes. However, only two of them, *TMEM16A* and *TMEM16B*, have unequivocally been shown to encode CaCCs. The precise function of the other family members is not yet fully elucidated (Duran and Hartzell, 2011; Kunzelmann *et al*., 2011; Huang *et al*., 2012a; Pedemonte and Galietta, 2014; Picollo *et al*., 2015). TMEM16A and TMEM16B channels appear to have somewhat distinct expression patterns and physiological roles. For example, the TMEM16A channel contributes to functions such as transepithelial Cl^−^ transport (e.g. Kunzelmann *et al*., 2012; Veit *et al*., 2012; Huang *et al*., 2012b) and smooth muscle contraction (e.g. Davis *et al*., 2010; Manoury *et al*., 2010; Thomas‐Gatewood *et al*., 2011; Heinze *et al*., 2014; Wang *et al*., 2015), whereas the TMEM16B channel is implicated in the control of sensory processes such as olfaction and vision (e.g. Stephan *et al*., 2009; Stohr *et al*., 2009; Hengl *et al*., 2010).

CaCCs have long been considered potential therapeutic targets for a variety of conditions (Pedemonte and Galietta, 2014). Until recently, CaCC pharmacology was restricted to compounds with limited specificity and relatively low potency. The fenamate class of compounds, including niflumic acid, flufenamic acid and mefenamic acid, inhibits CaCCs at micromolar concentrations. However, the fenamates also modulate other ion channels within a similar concentration range (Peretz *et al*., 2005; Gradogna and Pusch, 2010; Gwanyanya *et al*., 2012; Guinamard *et al*., 2013). Other commonly used CaCC blockers, such as 4,4′‐diisothiocyanato‐stilbene‐2,2′‐disulfonic acid (DIDS), 4‐acetamido‐4′‐isothiocyanatostilbene‐2,2′‐disulfonic acid (SITS) and tamoxifen, are much less potent (Hartzell *et al*., 2005; Huang *et al*., 2012a). Many of these CaCC inhibitors also reportedly interact with other non‐ion channel targets. Tamoxifen, for example, is an oestrogen antagonist (Jordan, 2006), while the fenamates serve as non‐steroidal anti‐inflammatory drugs by acting on COX (Mitchell and Warner, 1999). Furosemide, ethacrynic acid, mibefradil, glibenclamide, fluoxetine and mefloquine have a range of specific clinical uses and also inhibit CaCCs (Eggermont, 2004; Hartzell *et al*., 2005).

### TMEM16A inhibitors

High‐throughput screening enabled the identification of new TMEM16A inhibitors such as the relatively potent T16_inh_‐A01, (IC_50_ ≈ 1 μM) compound (Namkung *et al*., 2011a). Another screening study identified benzbromarone, dichlorophen and hexachlorophen as TMEM16A inhibitors (Huang *et al*., 2012b). Some natural compounds such as tannic acid, gallotannins and eugenol also appear to inhibit TMEM16A channels (Namkung *et al*., 2010; Yao *et al*., 2012). MONNA, a potent (IC_50_ ≈ 1 μM) TMEM16A inhibitor that does not interact with anion channels such as CFTR, ClC‐2 and bestrophin 1, has also been identified (Oh *et al*., 2013). However, the selectivity of T16_inh_‐A01 and MONNA has been questioned (Boedtkjer *et al*., 2015).

### TMEM16A activators

High‐throughput screening has also enabled the discovery of small molecules that promote TMEM16A channel activity such as a N‐aroylaminothiazole, termed E_act_, and a tetrazolylbenzamide, called F_act_. These compounds activated CaCC currents with an EC_50_ of ~3–6 μM (Namkung *et al*., 2011b). The selectivity and mechanism of action of these drugs have not yet been fully elucidated. However, E_act_ activated TMEM16A in the absence of intracellular Ca^2+^, which suggests that E_act_ has a Ca^2+^‐independent mechanism of action. In contrast, F_act_ appeared to potentiate the effect of Ca^2+^ (Namkung *et al*., 2011b).

### Anthracene‐9‐carboxylic acid (A9C): a drug with bimodal action

A9C , a compound which inhibits a variety of Cl^−^ channels (Zhou *et al*., 1997; Pusch *et al*., 2002; Estevez *et al*., 2003; Ai *et al*., 2004), has attracted substantial interest in the CaCC research community. Piper and Greenwood (2003) demonstrated an A9C‐mediated biphasic effect on native CaCCs (Piper and Greenwood, 2003). Opposing inhibiting and activating effects on the currents were also observed when cloned TMEM16A (Bradley *et al*., 2014) and TMEM16B channels (Cherian *et al*., 2015) were exposed to A9C. Key findings reported by Bradley *et al*. (2014) include inhibition of cloned human TMEM16A channels at positive *V*_*m*_ and slowing of current deactivation kinetics at negative *V*_*m*_. In this study, the potency of A9C block (IC_50_) was quantified only at a single positive *V*_*m*_ and the effects on current kinetics assessed in the presence of a single concentration of A9C. Another recent study (Reyes *et al*., 2015) determined the extent of channel block by extracellular A9C on cloned *Xenopus laevis* TMEM16A at various *V*_*m*_. However, the current kinetics at negative *V*_*m*_ was not examined. Additional useful insights were provided by the work of Cherian *et al*. (2015, who reported that extracellular A9C could inhibit TMEM16B channels that were maximally activated by [Ca^2+^]_i_. In the presence of lower [Ca^2+^]_i_, A9C inhibited TMEM16B currents at positive *V*_*m*_ and increased TMEM16B tail currents at negative *V*_*m*_ (Cherian *et al*., 2015).

Collectively, these existing published papers indicated that A9C acts as both an inhibitor and activator of TMEM16 channels. However, a number of factors limit the comparison and integration of these published results. These include the following: (i) the use of TMEM16 channels cloned from different species or different TMEM16 isoforms and (ii) the use of different experimental conditions (such as [Ca^2+^]_i_). Furthermore, these studies centre on the effects of extracellular A9C, while the effects of intracellular A9C application were not examined. Thus, we set out to perform a comprehensive analysis of A9C interaction with cloned mouse TMEM16A channels. Understanding the A9C mechanism of action is of considerable importance, particularly as A9C may constitute a novel drug template for the generation of specific TMEM16A channel blockers and activators.

This study employs various configurations of the patch‐clamp technique and a specific protocol designed to isolate A9C‐mediated activation from A9C inhibition. We found that A9C acted as an open channel blocker of cloned TMEM16A channels. A9C‐mediated activation was due to a hyperpolarizing shift of the steady‐state activation curves and slowing of deactivation kinetics. Both effects appeared to involve direct binding of A9C into the pore of TMEM16A channels to a region that is accessible from the extracellular side of the membrane.

## Methods

### Cell culture and transfection

Mouse TMEM16A (GenBank NM_178642) subcloned into pcDNA3.1 vector was used in this study (Alexander *et al*., 2013). HEK‐293T cells were cultured as previously described (Smith *et al*., 2013) and transfected with 0.5–1 μg of TMEM16A and 0.1 μg of CD8 constructs using Fugene HD (Promega, Madison, WI, USA) according to the manufacturer's instructions. Cells were used ~12–36 h after transfection. Transfected cells were visualized using the anti‐CD8 antibody‐coated beads method (Jurman *et al*., 1994).

### Electrophysiology

TMEM16A currents were measured with the whole‐cell or inside‐out patch configuration of the patch‐clamp technique as detailed in the Supporting Information. The exchange of solutions was achieved by using a local perfusion system consisting of eight tubes of 1.2 mm diameter in which the tip of the patch pipette was inserted. In some experiments, ultra‐rapid (<50 ms) changes in A9C concentration ([A9C]) (‘concentration jumps’) were achieved using a computer‐controlled perfusion system (Warner Instruments, Hamden, CT, USA).

### Composition of solutions

The intracellular solution contained (mM) 130 CsCl, 10 EGTA, 1 MgCl_2_, 10 HEPES and 8 CaCl_2_ to obtain ~300 nM of [Ca^2+^]_i_; pH was adjusted to 7.3 with NaOH. The intracellular solutions containing ~600 nM, ~1000 nM and ~12.5 μM [Ca^2+^]_i_ were obtained by replacing EGTA with equimolar H‐EDTA and by adding 2.1, 3.1 and 7.8 mM CaCl_2_ respectively. The extracellular solution contained (mM) 150 NaCl, 1 CaCl_2_, 1 MgCl_2_, 10 glucose, 10 D‐mannitol and 10 HEPES; pH was adjusted to 7.4 with NaOH. In some experiments, NaCl was reduced to 30 mM, and D‐mannitol proportionally increased to ensure the osmolarity of extracellular solution was unchanged. A9C was dissolved in DMSO (stock concentration, 300 mM); thus, the final concentration of DMSO was ≤1%. Aliquots of the stock solution were kept at −20° and used within 5 days.

### Stimulation protocols

#### Current versus *V*_*m*_ relationship

Current versus *V*_*m*_ relationships were constructed by measuring currents in response to 1 s *V*_*m*_ steps (test pulses) from −100 to +100 mV in 40 mV increments (unless stated otherwise) after a 1 s *V*_*m*_ step to +70 mV (pre‐pulse). Pulses were elicited every 2 s from a holding *V*_*m*_ of 0 mV. Steady‐state currents were measured at the end of the test pulses. As detailed in the Results, in the presence of 300 nM [Ca^2+^]_i_ and extracellular A9C, the test‐pulse currents reached a peak within the first ~50 ms of the test pulse. These peak currents were also measured and plotted against the *V*_*m*_ of the test pulse. Current density was obtained by dividing the current amplitude for the cell capacitance.

For determination of the current reversal potential (*E*_*rev*_), instantaneous currents were estimated from extrapolation of single exponential fits of the test‐pulse currents to the beginning of each test pulse. These instantaneous current values were plotted as a function of the *V*_*m*_, and the relative chord conductance was measured between an interval of ±20 m V around the *E*_rev_ (Tammaro *et al*., 2005; Adomaviciene *et al*., 2013).

#### Current versus [A9C] relationship

The relationship between [A9C] and TMEM16A current inhibition was obtained by measuring the currents at the end of 250 ms *V*_*m*_ steps from +20 to +120 mV in 20 mV increments elicited every 2 s from a holding *V*_*m*_ of −70 mV. TMEM16A currents measured in the presence of A9C (*I*_*b*_) were normalized to currents measured in the absence of A9C (*I*_0_) and plotted against [A9C]. The concentration–response curves obtained were fit with the Hill equation of the form:

(1) $$\frac{I_{b}}{I_{o}}=\frac{1}{1+{\left(\frac{\left[A9C\right]}{K_{i}}\right)}^{\gamma }},$$

where *K*_*i*_ is the apparent A9C dissociation constant from the inhibitory site and *γ* is the coefficient of cooperativity (Hill coefficient).

The dose–response curves for activation by A9C were fitted with a modified Hill equation of the form:

(2) $$\frac{I_{a}}{I_{o}}=1+\frac{A_{\max}-1}{1+{\left(\frac{\left[A9C\right]}{K_{d}}\right)}^{h}},$$

where *I*_*a*_ is the current in the presence of A9C, *A*_*max*_ is the maximal current activation, *K*_*d*_ is the apparent A9C dissociation constant from the activating site and *h* is the coefficient of cooperativity (Hill coefficient).

The relationships between the *K*_*i*_ or *K*_*d*_ and *V*_*m*_ were fitted with the Woodhull equation (Woodhull, 1973):

(3) $$K_{x}=K_{x\left(0\right)}\exp\left(\frac{-\delta_{x}\mathrm{zF}V_{m}}{\mathrm{RT}}\right).$$

The subscript ‘*x*’ stands for either ‘*i*’ or ‘*d*’. Thus, *K*_*i*(0)_ or *K*_*d*(0)_ are the apparent A9C dissociation constants from the inhibitory or activating site at 0 mV, *z* is the A9C electric valence (−1), *δ*_*i*_ or *δ*_*d*_ is the fraction of the *V*_*m*_ sensed by A9C when bound to the inhibitory or activating site, *F* is Faraday's constant, *R* is the universal gas constant and *T* is the absolute temperature.

#### Noise analysis

For non‐stationary noise analysis (Heinemann and Conti, 1992; Tammaro and Ashcroft, 2007), 80–200 identical pulses to a test potential of −70 mV (filtered at 10 kHz and sampled at 50 kHz) were applied, and the mean response, *I*, was calculated. The variance, *σ* ^2^, was computed from the average squared difference of consecutive traces. Background variance and current at 0 mV were subtracted, and the variance–mean plot was fit by

(4) $$\sigma^{2}=\mathrm{iI}-\frac{I^{2}}{N},$$

with the single channel current, *i*, and the number of channels, *N*, as free parameters.

TMEM16A channels are almost instantaneously activated by changes in *V*_*m*_ in the presence of high [Ca^2+^]_i_ (12.5 μM) (e.g. Adomaviciene *et al*., 2013). The lack of slow time‐dependent changes in current amplitude under these conditions makes non‐stationary noise analysis unfeasible. Thus, for experiments carried out in high [Ca^2+^]_i_, stationary noise analysis was performed instead. In the absence or presence of a fixed [A9C]_ext_, 10 tracts (each 600 ms in duration) of stationary currents measured at +70 mV were low‐pass filtered at 10 kHz and sampled at 50 kHz. For each tract of current, the variance and mean were calculated. The variance–mean plots obtained in the absence and presence of various [A9C]_ext_ were fitted simultaneously with Equation (4).

#### Steady‐state activation curve

The voltage‐dependence of TMEM16A channels was assessed by constructing conductance (*G*) versus *V*_*m*_ relationships in the presence of various [Ca^2+^]_i_. A 1 s pre‐pulse applied to different *V*_*m*_ (from −100 to +180 mV in 40 mV increments) was followed by a 0.5 s tail pulse to −60 mV. These pulses were elicited every 2 s from a holding *V*_*m*_ of 0 mV. Tail currents were fitted with a single exponential function and the instantaneous tail current amplitude (*I*_*tail*_) estimated from extrapolation of the fit to the beginning of the tail pulse. *G* was calculated as *G* = *I*_*tail*_/(*V*_*m*_ *− E*_*Cl*_), and normalized *G* (*G*/*G*_*max*_) was plotted against the *V*_*m*_ of the pre‐pulse. The *G* versus *V*_*m*_ relationships were fitted with the Boltzmann equation of the following form:

(5) $$\frac{G}{G_{\max}}=\frac{1}{1\;+\;\exp\left[\frac{z_{g}\left(V_{0.5}-\;V\right)F}{\mathrm{RT}}\right]},$$

where *z*_*g*_ is the number of gating charges moving through the applied transmembrane electric field during channel activation, and *V*_0.5_ is the voltage at which the *G* is half‐maximal and depends on the conformational energy required for the channel to open.

#### Data analysis

Data were analysed with routines developed by Dr P. Tammaro in the IgorPro (Wavemetrics, OR, USA) environment. Student's two‐tailed *t*‐test or anova with Bonferroni's *post* test were used for statistical analysis as appropriate, and *P* < 0.05 was considered significant. Data are given as mean ± SEM alongside the number of experiments (*n*).

## Results

### Effects of A9C on cloned TMEM16A channel currents

#### Current amplitude

**Figure 1:**
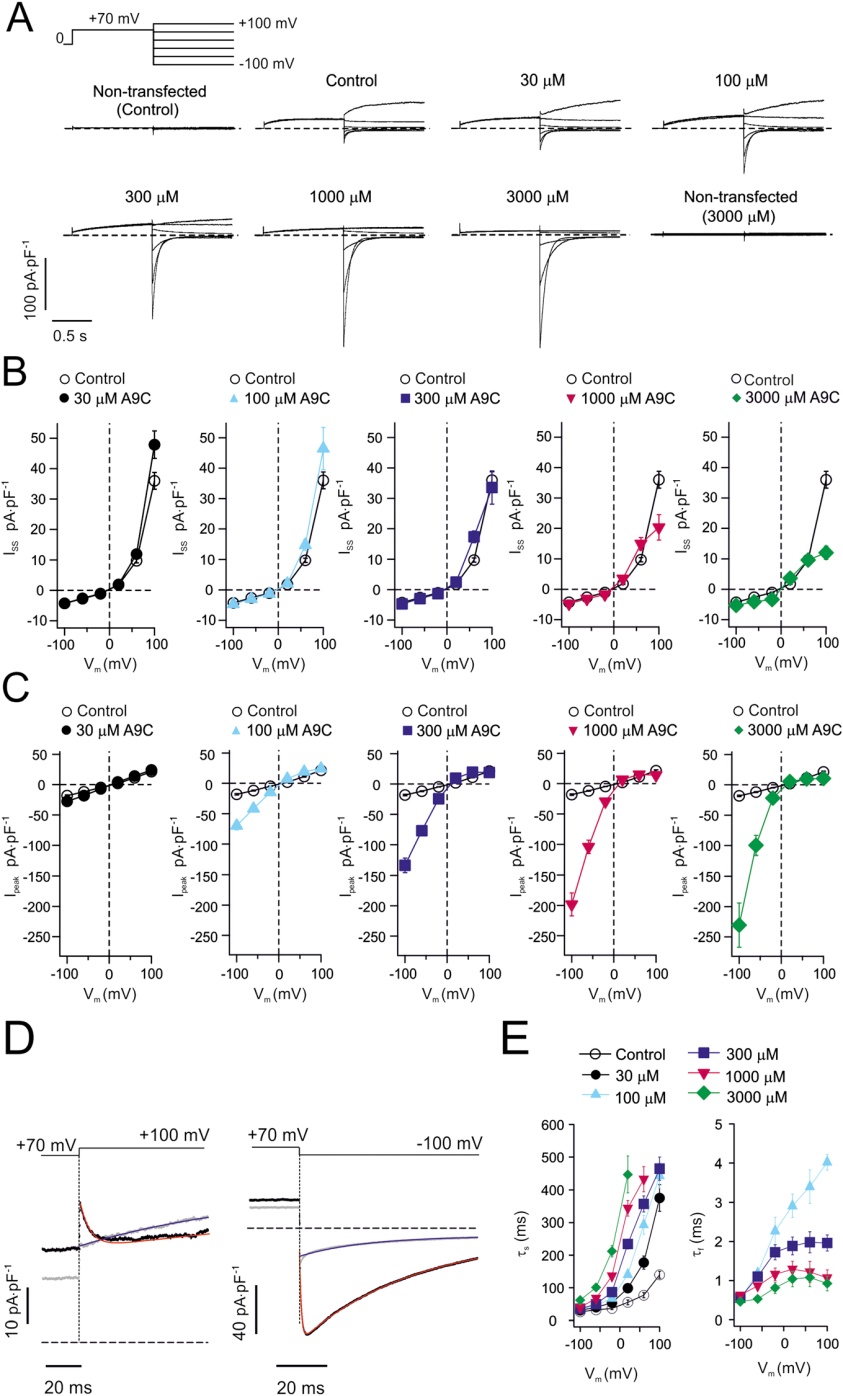
Effects of extracellular A9C on whole‐cell TMEM16A current amplitude and kinetics. (A) Whole‐cell currents recorded from non‐transfected HEK‐293T cells or from a HEK‐293T cell expressing TMEM16A in the absence (control) or presence of various [A9C]_ext_, as indicated. [Ca^2+^]_i_ was 300 nM. Stimulation protocol is shown in upper left panel. Dashed horizontal lines represent the zero‐current level. (B) Mean steady‐state whole‐cell current density versus *V*_*m*_ relationships measured in the presence of various [A9C]_ext_, as indicated. Control data are presented in the left‐most panel and re‐plotted in all subsequent panels. The number of experiments was 13 in each case. (C) Mean peak whole‐cell current density versus *V*_*m*_ relationships measured in the presence of various [A9C]_ext_ as indicated. Control data are presented in the left‐most panel and re‐plotted in all subsequent panels. The number of experiments was 13 in each case. (D) Whole‐cell currents recorded from a HEK‐293T cell expressing TMEM16A in response to the stimulation protocol shown in the panel above each trace. [Ca^2+^]_i_ was 300 nM. Recordings were taken in the absence (grey) or presence (black) of 300 μM [A9C]_ext_. The continuous coloured line represents the fit of the current traces with single (blue) or double (red) exponential functions. (E) Mean *τ*_*s*_ and *τ*_*f*_ recorded in the absence (control) or presence of various [A9C]_ext_, as indicated. The number of experiments was 13 in each case.

Previous reports indicated that A9C binds native CaCCs primarily from the extracellular side of the membrane (Qu and Hartzell, 2001). The project began by examining if this also applies to cloned TMEM16A channels. In the presence of increasing extracellular [A9C] ([A9C]_ext_), a pre‐pulse of +70 mV was used to open TMEM16A channels followed by a series of test pulses (Figure 1).

As previously reported (e.g. Schroeder *et al*., 2008; Xiao *et al*., 2011; Adomaviciene *et al*., 2013), in the presence of 300 nM [Ca^2+^]_i_, HEK‐293T cells transfected with TMEM16A displayed a prominent outwardly rectifying steady‐state current–*V*_*m*_ relationship when the *V*_*m*_ was stepped from −100 to +100 mV (Figure 1). The steady‐state current–*V*_*m*_ relationship varied in a complex manner as [A9C]_ext_ was varied between 30 and 3000 μM (Figure 1). For [A9C]_ext_ ≤ 100 μM and at *V*_*m*_ > 60 mV, the steady‐state current increased by ~1.3‐folds (Figure 1B). At higher [A9C]_ext_, however, the current at these same *V*_*m*_ decreased (by up to approximately threefold at 3000 μM [A9C]_ext_). The steady‐state current at *V*_*m*_ ≤ 60 mV did not change substantially at any [A9C]_ext_ tested.

As described in greater detail below, in the presence of extracellular A9C, the current during the test pulse presented a biphasic time‐course and reached a peak within the first ~50 ms of the test pulse. As [A9C]_ext_ was increased, the peak current at negative *V*_*m*_ progressively increased (Figure 1). For example, in the presence of 3000 μM A9C, the current at −100 mV was ~13‐folds higher than currents measured at −100 mV in the absence of A9C (control).

To test the possibility that the changes in current amplitude described above were due to alterations in TMEM16A channel ion selectivity, the *E*_*rev*_ of TMEM16A currents in the absence and presence of 3000 μM [A9C]_ext_ was assessed (Supporting Information Figure 1). The *E*_*rev*_ was 4.5 ± 1.4 mV (*n* = 13, control) and 8.3 ± 1.4 mV (*n* = 13, 3000 μM [A9C]_ext_, *P* < 0.05). These values are very close to the expected *E*_*rev*_ for Cl^−^ in our recording conditions (~1 mV).

To test the possibility that A9C activated endogenous currents in HEK‐293T cells, the effect of [A9C]_ext_ (3000 μM) on non‐transfected cells was assessed. No significant increase in current was observed relative to the small endogenous currents measured in non‐transfected cells in the absence of [A9C]_ext_ (Figure 1A). For example, at +100 mV, the current recorded from non‐transfected HEK‐293T cells in the absence and presence of 3000 μM [A9C]_ext_ was 3.6 ± 0.8 pA∙pF^−1^ (*n* = 6) and 3.7 ± 0.7 pA∙pF^−1^ (*n* = 6) respectively.

#### Current kinetics

In the absence of A9C, the test‐pulse current kinetics were well fitted with a single‐exponential function (Figure 1D). The time constant (*τ*_*s*_) arising from this single‐exponential fit increased as *V*_*m*_ was increased from −100 to +100 mV. For example, in the absence of A9C, *τ*_*s*_ was 26 ± 2 ms (*n* = 13) at −100 mV compared with 140 ± 10 ms (*n* = 13) at +100 mV (*P* < 0.05; Figure 1E). In contrast, in the presence of [A9C]_ext_ > 30 μM, the time‐course of the current during the test pulse followed the sum of two exponentials. At positive *V*_*m*_, the current first decreased in absolute amplitude with an exponential time‐course with time constant *τ*_*f*_ (Figure 1). This was followed by an exponential increase in current amplitude, with a time constant *τ*_*s*_ (Figure 1D). At negative *V*_*m*_, the trend was opposite: the current first increased in absolute amplitude followed by a decline to a new steady‐state value (Figure 1D). For *V*_*m*_ greater than −100 mV, as [A9C]_ext_ was increased, *τ*_*s*_ increased and *τ*_*f*_ decreased (Figure 1E).

Thus, the first set of experiments indicated that extracellular A9C produced complex effects on TMEM16A currents. The main changes being (i) activation of the peak currents measured at the beginning of the test pulse at negative *V*_*m*_ and (ii) current inhibition, observed predominantly at positive *V*_*m*_.

#### Characterization A9C‐mediated TMEM16A channel inhibition

**Figure 2:**
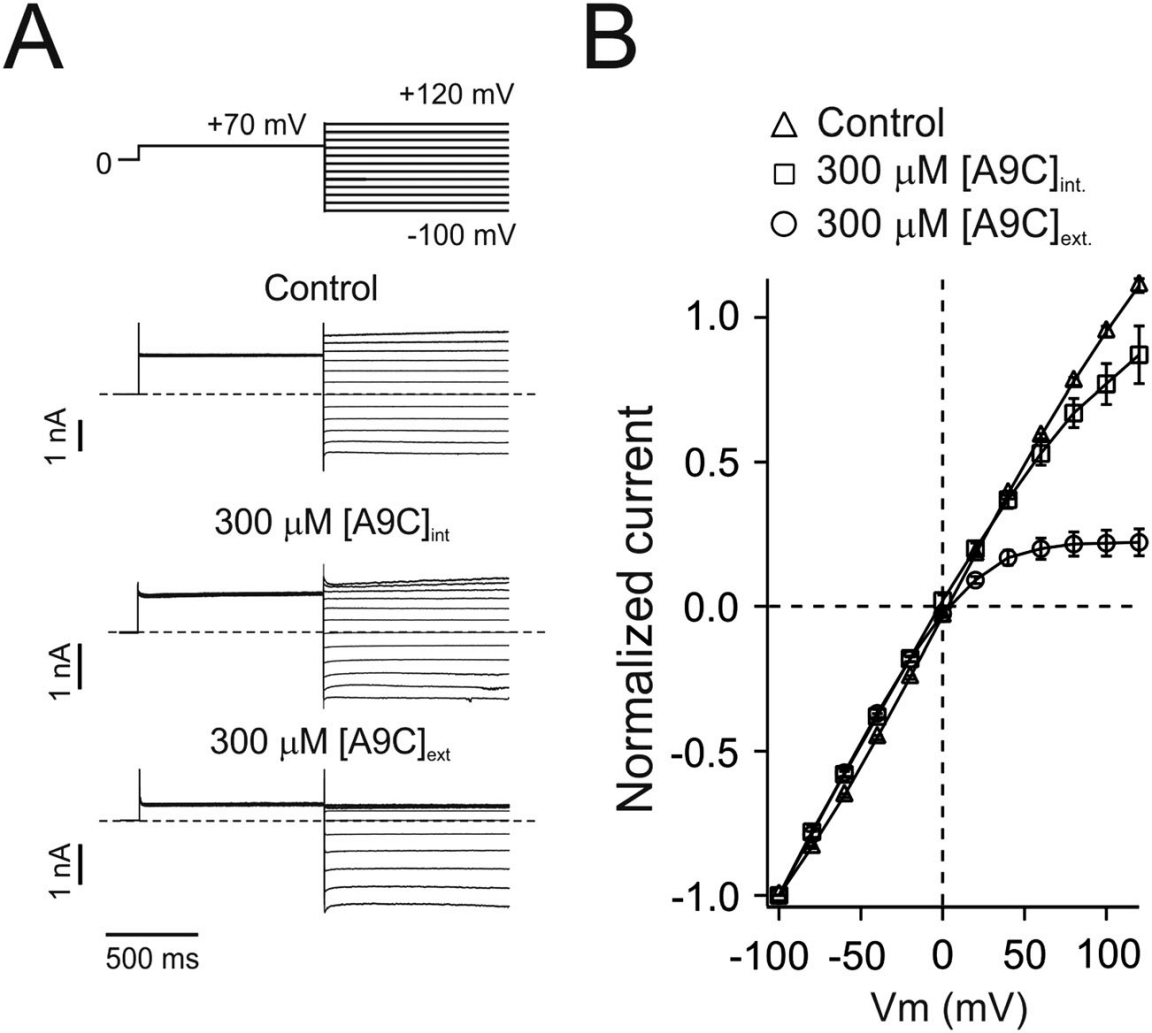
Effects of extracellular and intracellular A9C on the degree of rectification of TMEM16A current. (A) Currents recorded from inside‐out patches excised from HEK‐293T cells expressing TMEM16A. Currents were recorded in the absence (control) or presence of 300 μM [A9C], applied intracellularly or extracellularly, as indicated. [Ca^2+^]_i_ was 12.5 μM. Stimulation protocol is shown in the top panel. Dashed horizontal lines represent the zero‐current level. (B) Mean steady‐state current measured at the test pulse plotted against the test pulse *V*_*m*_. Currents were recorded in the absence or presence of 300 μM [A9C] applied intracellularly or extracellularly, as indicated. To allow visual comparison, currents were normalized for the current measured at −100 mV. The number of experiments was 6–16 in each case.

To study A9C block in isolation from the A9C activating effects on TMEM16A, currents were measured in the presence of 12.5 μM [Ca^2+^]_i_ in the inside‐out patch‐clamp configuration. High [Ca^2+^]_i_ was used to maximally activate TMEM16A channels. Consistent with previous studies (e.g. Schroeder *et al*., 2008; Xiao *et al*., 2011; Adomaviciene *et al*., 2013), in the presence of high [Ca^2+^]_i_, the TMEM16A current–*V*_*m*_ relationship measured at the end of 1 s test pulses from −100 to +120 mV was almost linear (Figure 2). When A9C (300 μM) was applied, the current–*V*_*m*_ relationship became inwardly rectifying. However, currents elicited at positive *V*_*m*_ were more markedly diminished in the presence of extracellular A9C compared with when A9C was applied to the intracellular side of the membrane. These effects were quantified by measuring the ratio of the steady‐state current measured at −100 and +120 mV (rectification index, I_−100_/I_+120_). In the absence of A9C, the I_−100_/I_+120_ was equal to 0.9 ± 0.1 (*n* = 16), while I_−100_/I_+100_ was 5.3 ± 1.0 (*n* = 6) and 1.2 ± 0.1 (*n* = 6) in the presence of 300 μM extracellular and intracellular A9C respectively.

Thus, A9C appeared to predominantly block TMEM16A channels from the extracellular side of the membrane at positive *V*_*m*_.

#### Extracellular A9C block of TMEM16A channels examined according to the Woodhull formalism

**Figure 3:**
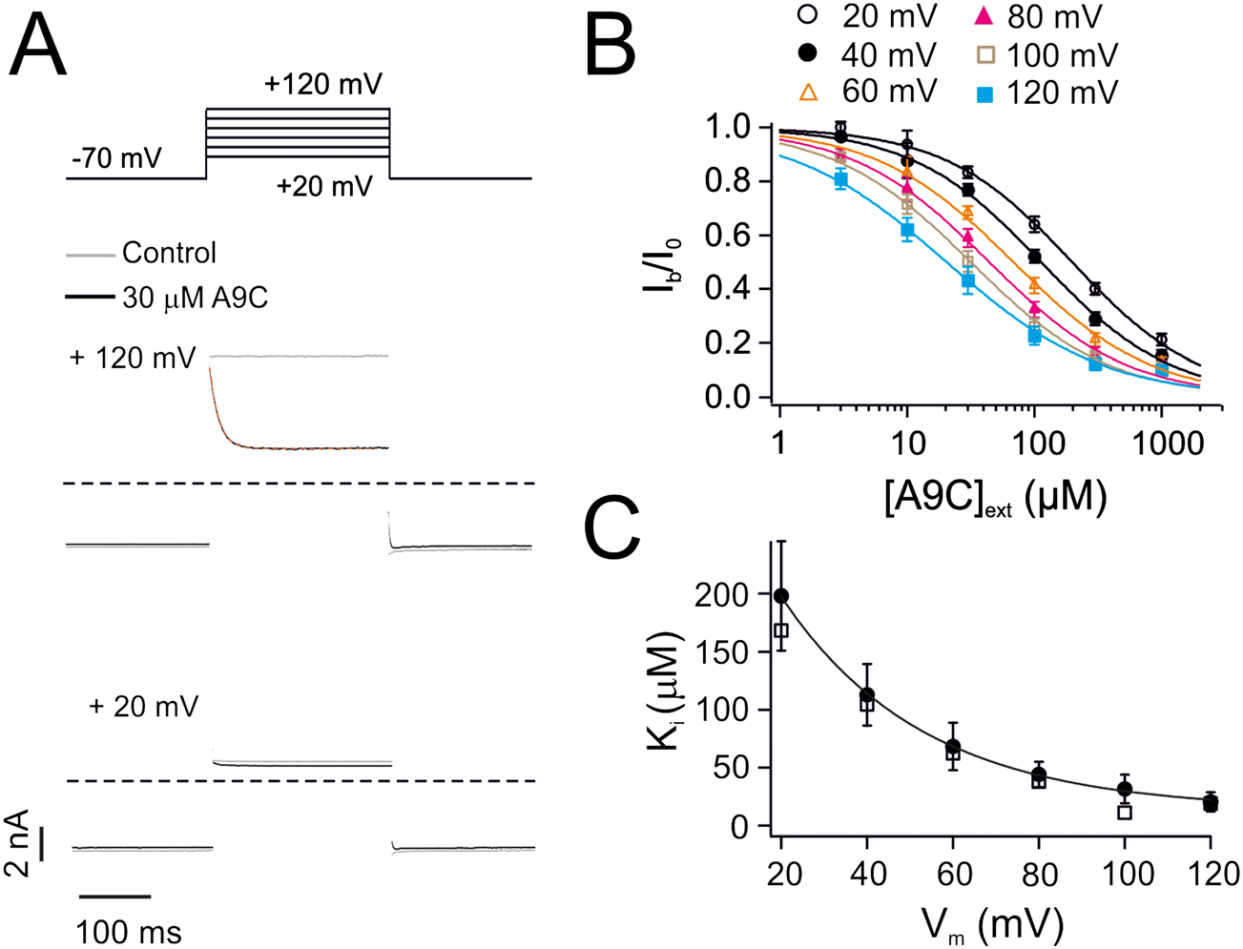
Extracellular A9C blocks TMEM16A channel via an open‐channel block mechanism. (A) Whole‐cell currents recorded from a HEK‐293T cell expressing TMEM16A in response to a 250 ms step to +20 mV or +120 mV, as indicated. The holding *V*_*m*_ was −70 mV. The complete stimulation protocol is shown in the top panel. [Ca^2+^]_i_ was 12.5 μM. Recordings were taken in the absence (grey) or presence (black) of 30 μM [A9C]_ext_. The broken red line represents the fit of the current with Supporting Information Equation (3). (B) Mean relationships between the [A9C]_ext_ and whole‐cell steady‐state currents normalized to the currents obtained in the absence of A9C (*I*_*b*_/*I*_0_) recorded at various *V*_*m*_, as indicated (*n* = 8). The smooth curves are the best fits of the data with Equation (1). (C) Filled symbols represent mean *K*_*i*_ (obtained from the Hill fit of the data showed in B) versus *V*_*m*_ relationship (*n* = 8). The smooth curve is the best fit of the data with Equation (3). Open symbols represent *K*_*i*_ calculated according to a Langmuir equation as described in the Supporting Information.

To investigate the precise mechanism by which extracellular A9C inhibits TMEM16A channels, macroscopic whole‐cell currents were recorded in the presence of 12.5 μM [Ca^2+^]_i_ and in response to 250 ms steps to various *V*_*m*_ test pulse from a holding *V*_*m*_ of −70 mV. The negative holding *V*_*m*_ was used to prevent channel block before the test pulse was elicited. Figure 3 shows that in the absence of A9C, when the *V*_*m*_ was stepped to a positive value, an instantaneous current was revealed that did not change in amplitude throughout the duration of the test pulse. In contrast, in the presence of A9C, the current during the test pulse rapidly decreased to a new steady‐state level. The time‐course of this decrease was described by a single‐exponential function characterized by a time constant *τ*_*B*_. The extent of the decrease in current was measured for various [A9C]_ext_ and various *V*_*m*_ test pulse and then plotted relative to the current measured in the absence of A9C (*I*_0_) (Figure 3B). The fit of these concentration–response relationships with Equation (1) yielded values of *K*_*i*_ and *γ* reported in Table 1. The *γ* values were close to 1 at each *V*_*m*_. In contrast, *K*_*i*_ decreased as the *V*_*m*_ was increased (Figure 3C). The relationship between *K*_*i*_ and *V*_*m*_ was fitted with Equation (3) with *δ*_*i*_ = 0.62 ± 0.03 (*n* = 8) and *K*_*i*(0)_ = 322 ± 13 μM (*n* = 8). Thus, A9C appeared to block TMEM16A channels according to a Woodhull mechanism, consistent with a single A9C molecule blocking the channel pore.

**Table 1:** Parameters (*K*_*i*_ and *γ*) obtained from the Hill fit of [A9C]_ext_‐TMEM16A inhibition relationships at various *V*_*m*_ in the presence of 12.5 μM [Ca^2+^]_i_

*V*_*m*_	20	40	60	80	100	120
*K*_*i*_	198 ± 47 (*n* = 8)	113 ± 26 (*n* = 8)	68 ± 20 (*n* = 8)	44 ± 10 (*n* = 8)	31 ± 12 (*n* = 8)	20 ± 8 (*n* = 8)
*γ*	0.86 ± 0.14 (*n* = 8)	0.85 ± 0.15 (*n* = 8)	0.8 ± 0.2 (*n* = 8)	0.8 ± 0.17 (*n* = 8)	0.8 ± 0.23 (*n* = 8)	0.71 ± 0.16 (*n* = 8)

*V*_*m*_, membrane potential; *K*_*i*_, inhibitory constant; *γ*, slope factor.

The *K*_*i*_ at various *V*_*m*_ can also be estimated from analysis of the time‐course of current inhibition at various *V*_*m*_ (see Supporting Information for details). *K*_*i*_ estimated from this analysis were plotted against the *V*_*m*_ in Figure 3C (open symbols). It is clear that *K*_*i*_ estimated in this way is similar to the *K*_*i*_ estimated from the Hill fit of the dose–response curves (filled symbols).

#### A9C block results in an apparent decline in TMEM16A channel *P*_*o*_

A macroscopic ionic current (*I*) through a specified number (*N*) of identical and independent ion channels is the product of the following:

(6) $$I=\mathrm{iN}P_{o}.$$

Thus, A9C block should manifest as a decrease in at least one of the parameters in this equation. A9C‐mediated current inhibition occurred in a sub‐second timescale. It is therefore reasonable to assume that *N*, the number of functional channels expressed in the plasma membrane, would remain unchanged as channel trafficking and expression usually occur over a longer timescale.

Stationary noise analysis was used to determine if A9C block manifests as a change in *i* and/or *P*_*o*_ of TMEM16A. Experiments were conducted in the presence of 12.5 μM [Ca^2+^]_i_ to enable examination of the effect of A9C block in the absence of A9C‐mediated activation. Mean current and variance were measured in tracts of stationary currents recorded in the absence or presence of different [A9C]_ext_.

Note that it follows from Equations 4 and 6 that

(7) $$\frac{\sigma^{2}}{I}=\;i\left(1-P_{o}\right).$$

**Figure 4:**
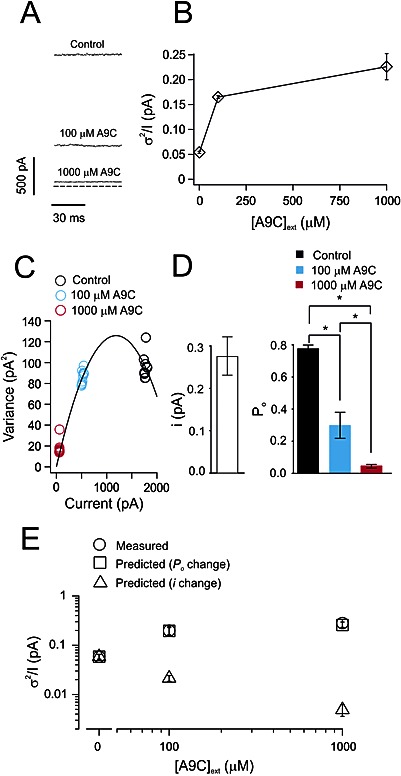
Stationary noise analysis for whole‐cell TMEM16A currents in the absence and presence of A9C block. (A) Whole‐cell currents recorded from a HEK‐293T cell expressing TMEM16A in the absence (control) or in the presence of different [A9C]_ext_, as indicated. [Ca^2+^]_i_ was 12.5 μM, and *V*_*m*_ was +70 mV. Horizontal dashed line represents the zero‐current level. (B) Mean *σ* ^2^/*I* obtained in the presence of different [A9C]_ext_. For each [A9C]_ext_, the *σ* ^2^/*I* obtained from 10 tracts of current were averaged. These data refer to the specific experiment shown in C. (C) Current variance plotted against the mean current for individual tracts of stationary currents recorded from a single TMEM16A‐expressing HEK‐293T cell in the presence of different [A9C]_ext_, as indicated. The parabolic line is the best simultaneous fit of the data using Equation (4). (D) Mean *i* and *P*_*o*_ obtained from stationary noise analysis. Number of experiments was 5 in each case. *P < 0.05. (E) Measured and predicted *σ* ^2^/*I* values plotted versus [A9C]_ext_.

*σ* ^2^/*I* for the tracts of stationary currents recorded in the absence or presence of different [A9C]_ext_ were calculated, averaged and plotted against [A9C]_ext_ (Figure 4B). It is clear that *σ* ^2^/*I* increases as [A9C]_ext_ increases. According to Equation (7), this trend is consistent with either an increase in *i* or an increase in (1 − *P*_*o*_). Note that it is unlikely that A9C block resulted in an increase in *i*, because open channel blockers usually provoke either an apparent decrease in *i* or leave *i* unaltered. Thus, the most likely possibility is that the underlying cause of macroscopic current inhibition by A9C is a reduction in channel *P*_*o*_ (hence an increase in (1 − *P*_*o*_)).

With this assumption in mind, the variance–mean plots were fitted with Equation (4) (Figure 4C). The fits yielded a single value of *i* of 0.27 ± 0.04 pA (*n* = 5). *P*_*o*_ values arising from the fits were 0.78 ± 0.02 for control (*n* = 5), 0.30 ± 0.08 at 100 μM [A9C]_ext_ (*n* = 5) and 0.04 ± 0.01 at 1000 μM [A9C]_ext_ (*n* = 5) (Figure 4D).

To confirm that the assumption described above was correct, the following calculations were performed. Theoretical *σ* ^2^/*I* in various [A9C]_ext_ were computed using Equation (7) assuming that either *i* or *P*_*o*_ were affected by [A9C]_ext_ by a fraction corresponding to the extent of macroscopic current inhibition. These calculated *σ* ^2^/*I* values were plotted against [A9C]_ext_ in Figure 4E. It is clear that the experimental data and calculations made under the assumption that *P*_*o*_ varies as a result of extracellular A9C application were comparable. In contrast, the calculations based on a change in *i* significantly deviated from the experimental results.

#### Characterization A9C‐mediated TMEM16A channel activation

**Figure 5:**
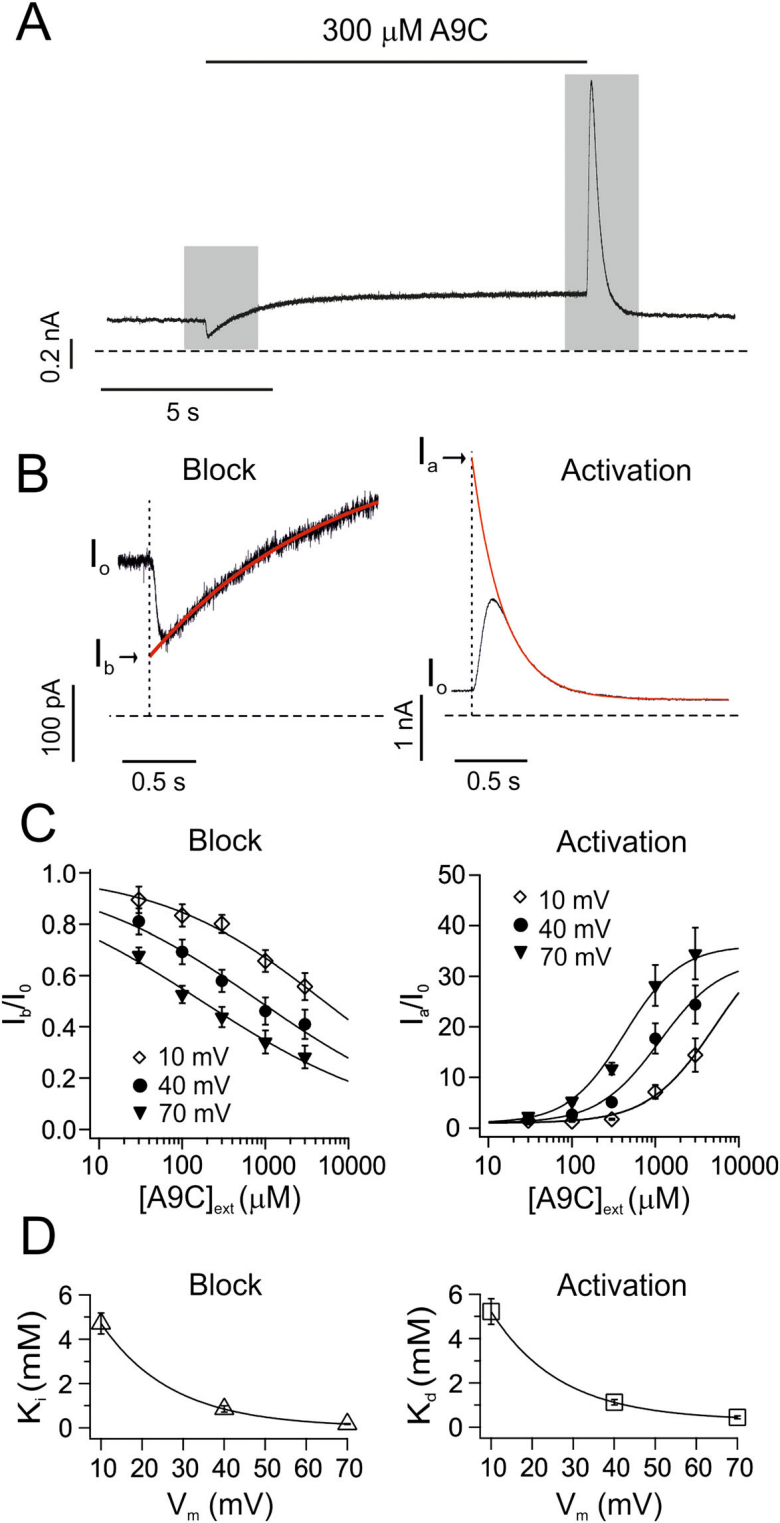
Concentration‐jump protocol to distinguish A9C‐mediated block and activation of TMEM16A channels. (A) Whole‐cell currents recorded from a HEK‐293T cells expressing TMEM16A. *V*_*m*_ was +70 mV. [Ca^2+^]_i_ was 300 nM. Extracellular A9C (300 μM) was applied as indicated by the horizontal bar via a fast‐perfusion system. The dashed horizontal line represents the zero‐current level. The shaded grey bars indicate regions of the recording that were expanded in B. (B) Expanded view of the currents highlighted by the grey bars shown in A. The continuous red traces represent single exponential fits used to back‐extrapolate the currents to obtain *I*_*b*_ (left panel) and *I*_*a*_ (right panel). Dashed horizontal lines represent the zero‐current level. (C) Mean inhibition (*I*_*b*_/*I*_0_) and activation (*I*_*a*_/*I*_0_) plotted versus [A9C]_ext_ at various *V*_*m*_, as indicated. Continuous lines through points are the best fit of the data with Equation (1) (left panel) or Equation (2) (right panel). (D) Mean *K*_*i*_ and *K*_*d*_ (obtained from the Hill fit of the data shown in C) versus *V*_*m*_ relationships. The smooth curves are the best fit of the data with Equation (4). The number of experiments was 9–11 in each case.

To study the activating effect of extracellular A9C on TMEM16A currents in isolation from A9C‐mediated inhibition, the following stimulation protocol was used. Whole‐cell currents were measured in response to a constant *V*_*m*_ of +70 mV and in the presence of 300 nM [Ca^2+^]_i_. Under these recording conditions and in the absence of extracellular A9C, the TMEM16A current remained at a constant amplitude (Figure 5A). When A9C was rapidly applied to the bath solution (concentration jump), the current amplitude rapidly declined before increasing to a new steady‐state level (Figure 5A and 5B). As A9C was removed, a dramatic increase in current was observed followed by a rapid return of the current to the level measured in the absence of A9C (Figure 5A and 5B). We interpret these phenomena as the combination of an inhibiting (block) and activating effect of A9C on TMEM16A channels: the initial decline in current when A9C was applied is likely to represent open‐channel block by A9C, and the subsequent current potentiation is probably caused by a slower allosteric effect on channel gating caused by A9C binding. Rapid washout of A9C led to the fast relief of channel block, while the allosteric activating effect appeared to persist. Thus, the inhibitory effect of A9C was estimated by back‐extrapolating the current amplitude (*I*_*b*_) to the point when the drug was added. The current activation (*I*_*a*_) was measured via back‐extrapolation of the transient current spike observed upon drug washout.

Each cell was exposed to various [A9C]_ext_, and the extent of blockage at each concentration expressed as *I*_*b*_/*I*_0_. The extent of current activation at each [A9C]_ext_ was assessed as *I*_*a*_/*I*_0_. The average activating and inhibitory responses measured at +70 mV are shown in Figure 5C.

To determine if the activating effect of [A9C]_ext_ on the TMEM16A current is *V*_*m*_‐dependent, experiments similar to those described above were performed at constant *V*_*m*_ of +10 and +40 mV (Figure 5C). From the average data presented in Figure 5C and Table 2, it can be concluded that both inhibition and activation are *V*_*m*_‐dependent. Figure 5C shows the fit of the *K*_*i*_ or *K*_*d*_ versus *V*_*m*_ relationships with Equation (3) with *K*_*i*(0)_ = 8370 ± 372 μM (*n* = 9), *δ*_*i*_ = 0.68 ± 0.03 (*n* = 9), *K*_*d*(0)_ = 8945 ± 378 μM (*n* = 9) and *δ*_*d*_ = 0.66 ± 0.03 (*n* = 9).

**Table 2:** Parameters obtained from the Hill fit of [A9C]ext‐TMEM16A inhibition (a) or activation (b) relationship at various V*_m_* and in the presence of 300 nM [Ca^2+^]i

*V*_*m*_ (mV)	IC_50_ (a); EC50 (b) (μM)	*γ* (a); *h* (b)
(a)		
10	4712 ± 478 (*n* = 9)	0.4 ± 0.1 (*n* = 9)
40	849 ± 140 (*n* = 11)	0.4 ± 0.1 (*n* = 11)
70	171 ± 21 (*n* = 11)	0.4 ± 0.1 (*n* = 11)
(b)		
10	5226 ± 582 (*n* = 9)	1.1 ± 0.1 (*n* = 9)
40	1124 ± 126 (*n* = 11)	1.2 ± 0.1 (*n* = 11)
70	440 ± 69 (*n* = 11)	1.1 ± 0.1 (*n* = 11)

*V*_*m*_, membrane potential; IC_50_, concentration causing half‐maximal inhibition; *γ*, Hill coefficient (inhibition).

EC_50_, concentration causing half‐maximal activation; *h* , Hill coefficient (activation).

**Figure 6:**
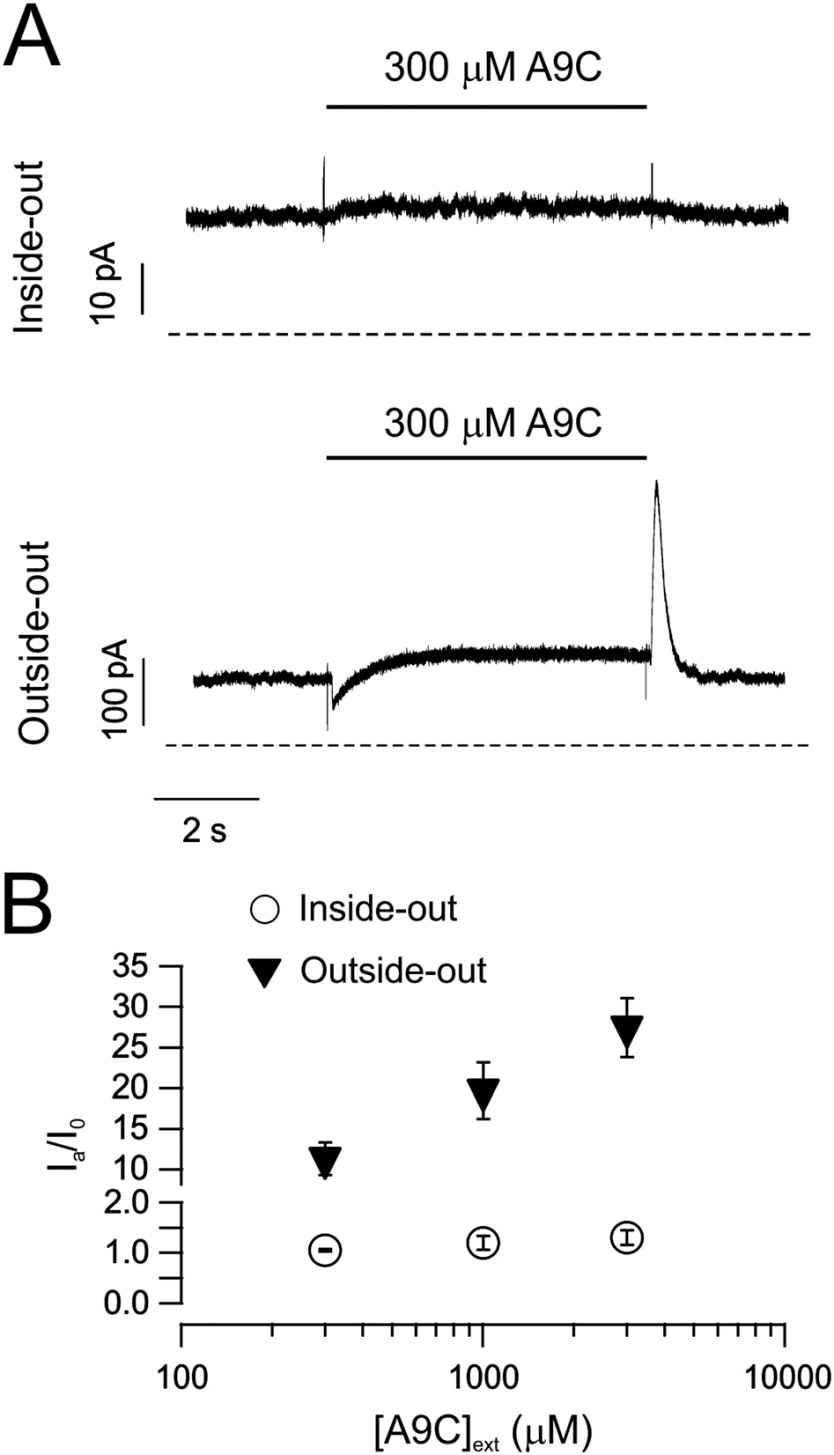
Activating effect of intracellular A9C on TMEM16A currents. (A) Currents recorded from an inside‐out or an outside‐out patch excised from HEK‐293T cells expressing TMEM16A. A9C (300 μM) was applied to the patch via a fast‐perfusion system as indicated by the horizontal bars. *V*_*m*_ was +70 mV. [Ca^2+^]_i_ was 300 nM. (B) Mean A9C‐mediated current increase (expressed as *I*_*a*_/*I*_0_) in response to intracellular A9C (inside‐out patches, *n* = 5) or extracellular A9C (outside‐out patches, *n* = 5).

To examine if intracellular A9C produced an activating effect on TMEM16A currents, concentration jump experiments were performed in the inside‐out configuration of the patch‐clamp technique, with A9C applied intracellularly and in the presence of 300 nM [Ca^2+^]_i_. Figure 6 shows that intracellular A9C produced only a small increase in the current in response to 300, 1000 and 3000 μM [A9C]. For example, *I*_*a*_/*I*_0_ in the presence of 3000 μM intracellular A9C during inside‐out patches was 1.3 ± 0.1 (*n* = 5), while extracellular A9C produced a *I*_*a*_/*I*_0_ of 27 ± 4 (*n* = 5) in outside‐out patches (Figure 6).

#### Competition with extracellular Cl^−^

**Figure 7:**
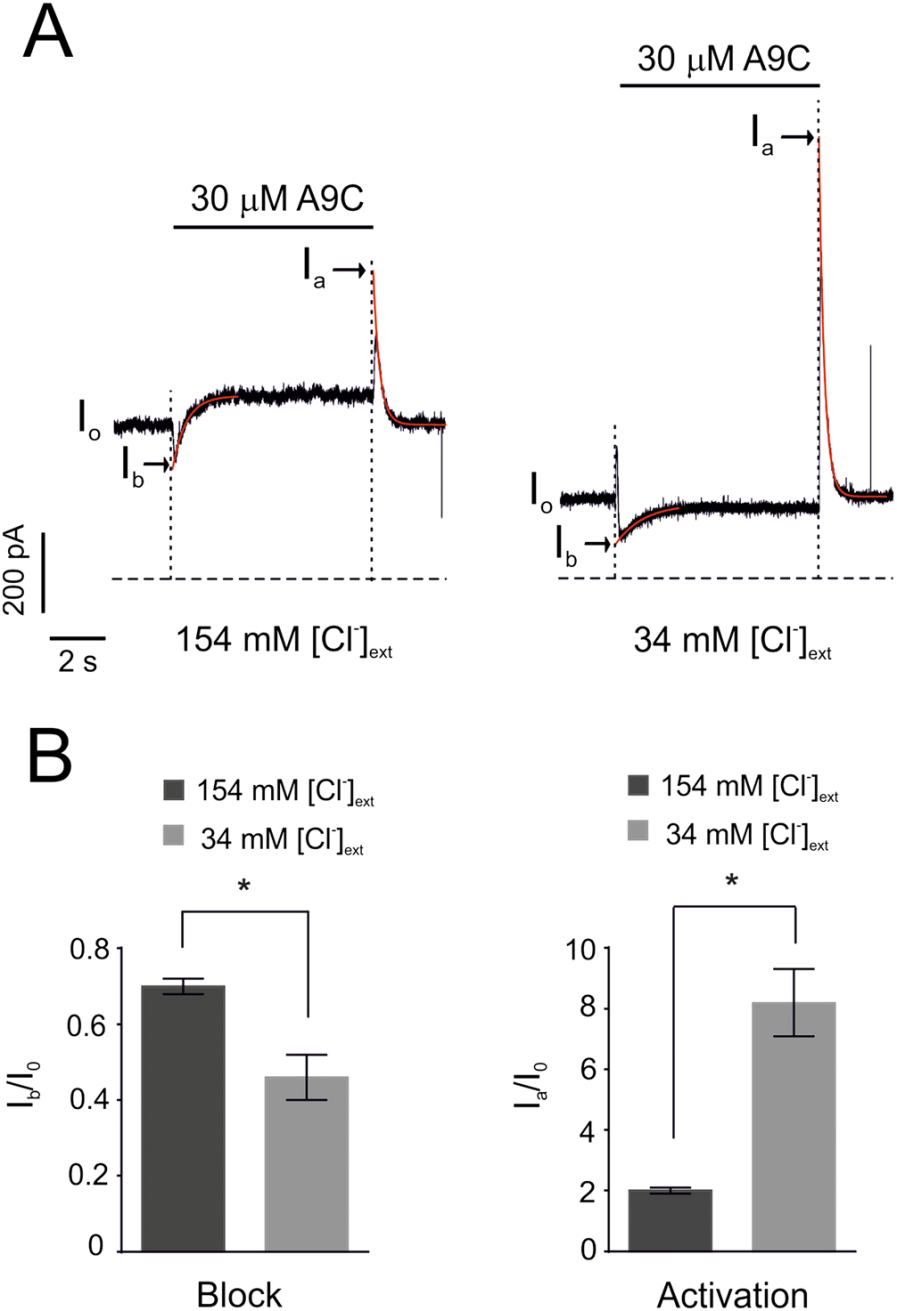
The effects of varying [Cl^−^]_ext_ on A9C‐mediated inhibition and activation of TMEM16A currents. (A) Whole‐cell currents recorded from a HEK‐293T cell expressing TMEM16A. Extracellular A9C (30 μM) was applied via a fast‐perfusion system as indicated by the horizontal bars. [Cl^−^]_ext_ was 154 mM (right panel) or 34 mM (left panel). The continuous red lines represent the single exponential functions used to back‐extrapolate the currents to obtain *I*_*b*_ and *I*_*a*_. The horizontal dashed lines represent the zero‐current level. (B) Mean extent of inhibition (*I*_*b*_/*I*_0_, left panel) and activation (*I*_*a*_/*I*_0_, right panel) of TMEM16A current in response to 30 μM [A9C]_ext_ and in the presence of different [Cl^−^]_ext_, as indicated. The number of experiments was seven in each case. *P < 0.05.

As outlined in the previous section, the current inhibition and activation induced by extracellular A9C presented a very similar degree of *V*_*m*_‐dependence. This suggests that these effects require A9C to bind to a site(s) located within the membrane‐spanning region of the channel, likely to be the permeation pathway. To test this possibility, the extent of TMEM16A current inhibition and activation were tested in the presence of high (154 mM) or low (34 mM) extracellular Cl^−^ concentrations ([Cl^−^]_ext_) (Figure 7). These experiments were conducted at a fixed *V*_*m*_ (+70 mV) and [Ca^2+^]_i_ (300 nM). In seven separate experiments, in the presence of low [Cl^−^]_ext_, application of 30 μM [A9C]_ext_ led to a fractional inhibition (*I*_*b*_/*I*_0_) of 0.46 ± 0.06 (*n* = 7). This contrasted with an *I*_*b*_/*I*_0_ of 0.70 ± 0.02 (*n* = 7) observed in high [Cl^−^]_ext_ (*P* < 0.05). The *I*_*a*_/*I*_0_ for activation was 8.2 ± 1.1 (*n* = 7) and 2.0 ± 0.1 (*n* = 7) in low and high [Cl^−^]_ext_ respectively (*P* < 0.05). Thus, extracellular Cl^−^ antagonizes both the inhibiting and activating effects by a similar extent suggesting that these effects require binding of A9C into the pore of the channel.

#### Extracellular A9C does not affect TMEM16A single channel conductance but alters channel *P*_*o*_

The next series of experiments aimed to determine the mechanism that underlies the TMEM16A current activation produced by extracellular A9C. As outlined above, a change in current amplitude may be due to a change in *i*, *N* and/or *P*_*o*_. As stated above, because A9C‐mediated current activation occurred in a sub‐second timescale, it is reasonable to assume that *N* would remain unchanged throughout the duration of the experiment. Thus, we examined if extracellular A9C alters the TMEM16A channel *i* or *P*_*o*_.

**Figure 8:**
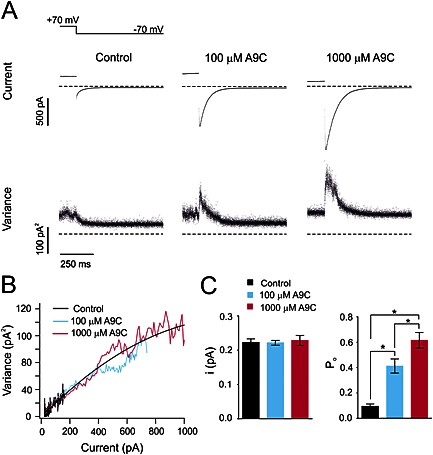
Non‐stationary noise analysis for whole‐cell TMEM16A current in the absence and presence of A9C activation. (A) Whole‐cell currents were recorded from a HEK‐293T cell expressing TMEM16A channels. Mean TMEM16A current and variance around the mean were obtained from 110 to 160 current traces recorded in response to a 1 s pulse to −70 mV (from a 1.5 s pre‐pulses to +70 mV). [Ca^2+^]_i_ was 300 nM. Horizontal dashed lines represent the zero‐current or zero‐variance level. Extracellular A9C was applied as indicated. (B) Current variance plotted against the mean current for the experiments shown in A. The parabolic line is the best fit of the data recorded in 1000 μM A9C using Equation (4). For clarity, fits of the data obtained in the absence (control) or in the presence of 100 μM [A9C]_ext_ are not shown. (C) Mean *i* and *P*_*o*_ obtained from non‐stationary noise analysis. Number of experiments was nine in each case. *P < 0.05.

Non‐stationary noise analysis was performed in the presence of 300 nM [Ca^2+^]_i_ during a tail pulse to −70 mV (Figure 8). In the absence of A9C, the *i* of TMEM16A was 0.22 ± 0.01 pA (*n* = 9). In the presence of 100 and 1000 μM [A9C]_ext_, *i* was 0.22 ± 0.01 pA (*n* = 9) and 0.23 ± 0.02 pA (*n* = 9) respectively. Thus, extracellular A9C did not affect the single TMEM16A channel current. In the absence of A9C, the *P*_*o*_ of TMEM16A was 0.09 ± 0.02 (*n* = 9) while in the presence of 100 and 1000 μM [A9C]_ext_, the *P*_*o*_ was 0.41 ± 0.06 (*n* = 9) and 0.62 ± 0.06 (*n* = 9) respectively. Thus, extracellular A9C increased TMEM16A channel *P*_*o*_ at a fixed *V*_*m*_ and [Ca^2+^]_i_.

#### Effects of A9C on the *V*_*m*_‐sensitivity and [Ca^2+^]_i_‐sensitivity of the TMEM16A channels

**Figure 9:**
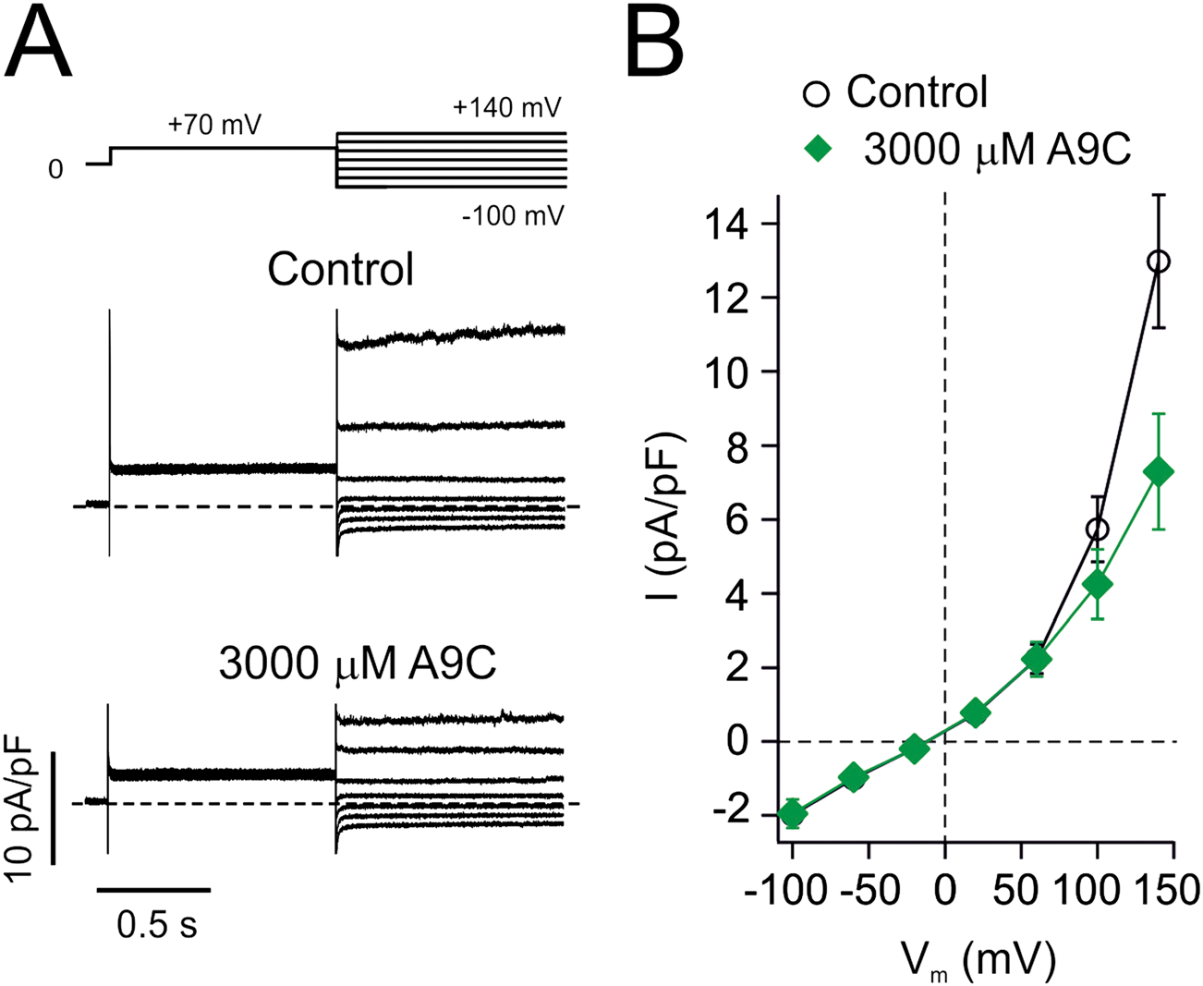
Effects of extracellular A9C on whole‐cell TMEM16A current amplitude in the absence of intracellular Ca^2+^. (A) Whole‐cell currents recorded from a HEK‐293T cell expressing TMEM16A in the absence (control) or presence of 3000 μM [A9C]_ext_, as indicated. [Ca^2+^]_i_ was 0. The stimulation protocol is shown in the top panel. Dashed horizontal lines represent the zero‐current level. (B) Mean steady‐state whole‐cell current density versus *V*_*m*_ relationships measured in the absence or presence of 3000 μM [A9C]_ext_, as indicated. The number of experiments was 5 in each case.

The gating of TMEM16A channels is modulated by changes in [Ca^2+^]_i_ and *V*_*m*_. To study the effect of A9C on the intrinsic *V*_*m*_‐dependence of the channel, extracellular A9C (3000 μM) was applied during whole‐cell patch‐clamp in the absence of [Ca^2+^]_i_ (Figure 9). The stimulation protocol was similar to that used in Figure 1. However, because TMEM16A current in the absence of [Ca^2+^]_i_ are evident at *V*_*m*_ greater than ~100 mV (Xiao *et al*., 2011), test pulses were elicited up to a value of +140 mV. Consistent with previous studies (Xiao *et al*., 2011), TMEM16A current in the absence of [Ca^2+^]_i_ appeared almost instantaneously in response to strong depolarizations (Figure 9). The TMEM16A current at +140 mV was 13.0 ± 4.0 pA∙pF^−1^ (*n* = 5). When extracellular A9C (3000 μM) was applied, the TMEM16A current was inhibited to 7.3 ± 3.8 pA∙pF^−1^ (*n* = 5), a value indistinguishable from the small endogenous current present in non‐transfected HEK‐293T cells (6.2 ± 0.5 pA∙pF^−1^ (*n* = 5) at +140 mV, not shown). In contrast to the effect of A9C in the presence of 300 nM [Ca^2+^]_i_ (Figure 1), there was no large increase in the tail currents at negative *V*_*m*_ in the absence of [Ca^2+^]_i_ (Figure 9). Thus, A9C activation does not involve changes to the intrinsic *V*_*m*_ sensitivity of TMEM16A channels.

**Figure 10:**
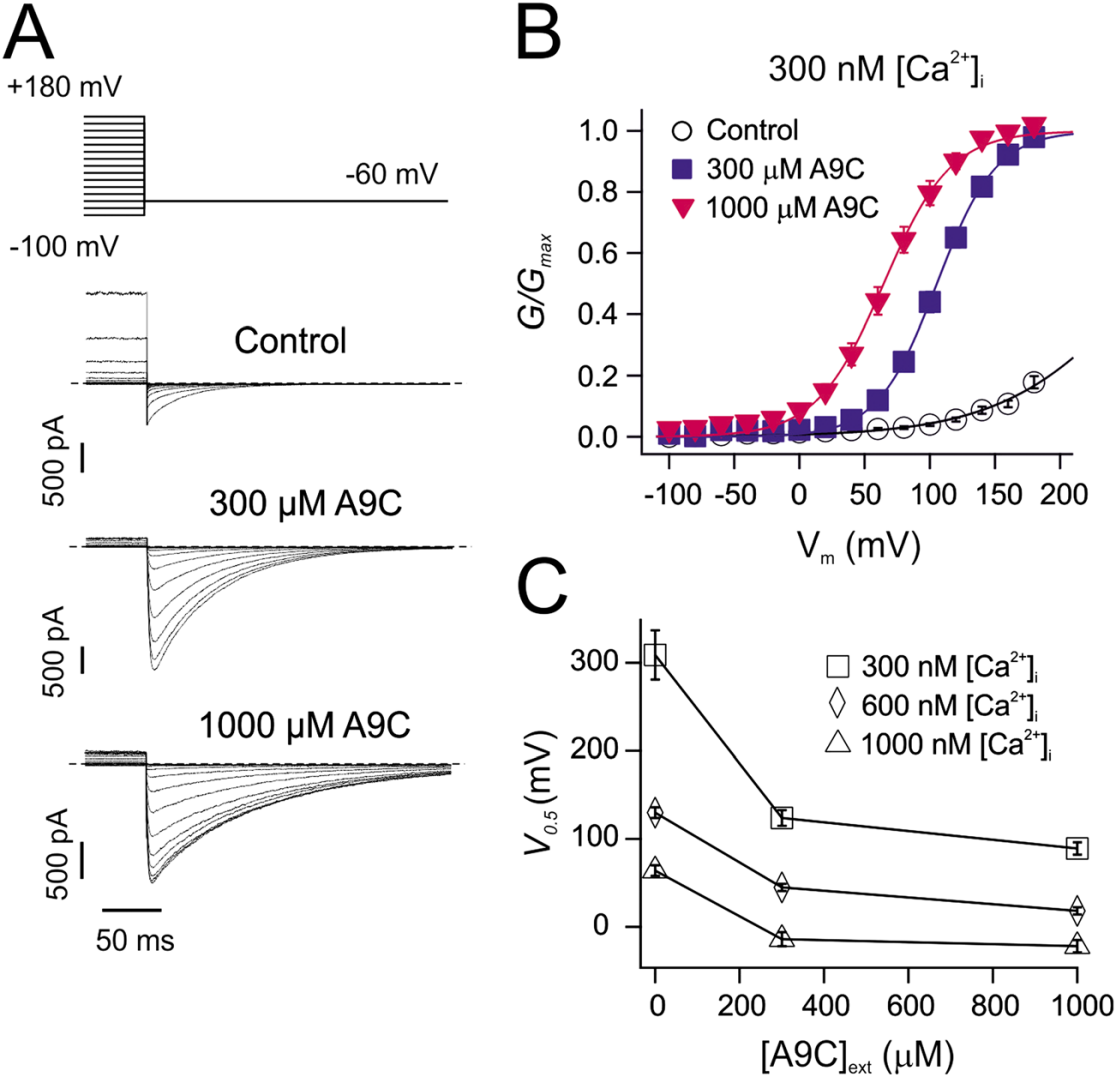
Voltage dependence of TMEM16A channels in the absence and presence of extracellular A9C. (A) Tail currents recorded from inside‐out patches excised from HEK‐293T cells expressing TMEM16A, in the presence of 300 nM [Ca^2+^]_i_ and various [A9C]_ext_, as indicated. Stimulation protocol is shown in the top panel. Horizontal dashed line indicates the zero‐current level. (B) Mean normalized TMEM16A conductance versus *V*_*m*_ relationships obtained in the presence of 300 nM [Ca^2+^]_i_ and various [A9C]_ext_, as indicated. (C) Mean *V*_0.5_ plotted versus [A9C]_ext_ measured in the presence of various [Ca^2+^]_i_, as indicated. The number of experiments was 5–14 in each case.

To examine the possible effects of A9C on TMEM16A channel [Ca^2+^]_i_‐sensitivity, the conductance versus *V*_*m*_ relationships were assessed at various [Ca^2+^]_i_ in the absence or presence of various [A9C]_ext_ (Figure 10 and Table 3). In the absence of extracellular A9C, *V*_0.5_ (derived from fit with Equation (5)) shifted progressively to lower values as the [Ca^2+^]_i_ was increased from 300 to 1000 nM (Table 3). Extracellular A9C dramatically shifted the steady‐state activation curves to the left (Figure 10 and Table 3) to a similar extent for all [Ca^2+^]_i_ tested.

**Table 3:** Parameters (*V*_0.5_ and *z*_*g*_) obtained from the Boltzmann fit of TMEM16A conductance versus *V*_*m*_ relationships at various [Ca^2+^]_i_ and [A9C]_ext_

[Ca^2+^]_i_ (nM)	Boltzmann parameters	[A9C]_ext_	300 μM [A9C]_ext_	1000 μM [A9C]_ext_
~300	*V*_0.5_, mV	309 ± 28 (*n* = 10)	124 ± 9 (*n* = 8)*	89 ± 7 (*n* = 14)*
	*z*_*g*_	1.4 ± 0.3 (*n* = 10)	0.7 ± 0.1 (*n* = 8)*	0.7 ± 0.1 (*n* = 14)*
~600	*V*_0.5_, mV	130 ± 6 (*n* = 10)	45 ± 4 (*n* = 8)*	18 ± 4 (*n* = 14)*
	*z*_*g*_	0.9 ± 0.1 (*n* = 10)	0.7 ± 0.1 (*n* = 8)*	0.7 ± 0.1 (*n* = 14)*
~1000	*V*_0.5_, mV	64 ± 6 (*n* = 10)	−15 ± 8 (*n* = 5)*	−22 ± 6 (*n* = 9)*
	*z*_*g*_	1.3 ± 0.1 (*n* = 10)	0.9 ± 0.1 (*n* = 5)*	0.9 ± 0.1 (*n* = 9)*

* Statistically significant difference from parameters obtained in the absence of A9C.

## Discussion

This study represents a comprehensive, mechanistic study of the effects of A9C on the TMEM16A channel. A9C inhibition occurred via an open channel blocking mechanism, whereas the activating effects were due to a dramatic increase in the apparent [Ca^2+^]_i_‐sensitivity of the channel. These effects primarily occurred when A9C was applied extracellularly and required binding of A9C to a site located within the channel pore.

### A9C as an open channel blocker

At physiological pH, A9C is a negatively charged molecule that is known to block the permeation pathway of many Cl^−^ channels including ClC‐1 (Estevez *et al*., 2003), CFTR (Ai *et al*., 2004), swelling‐activated Cl^−^ channels (Shuba *et al*., 2004) and CaCCs (Qu and Hartzell, 2001; Piper and Greenwood, 2003). Recently, Reyes *et al*. (2015) reported that A9C inhibited *Xenopus laevis* TMEM16A channels in a *V*_*m*_‐dependent manner with a *K*_*i*_ of ~90 μM at +80 mV. Bradley *et al*. (2014) estimated a *K*_*i*_ for human TMEM16A of ~60 μM at +80 mV. These values are close to the value we determined (~50 μM at +80 mV) for mouse TMEM16A. This suggests that the A9C binding site may be conserved among different species. Our study is the first to compare the potency of A9C block of TMEM16A at different [Ca^2+^]_i_. The *K*_*i*(0)_ in the presence of 300 nM [Ca^2+^]_i_ was ~8.4 mM, whereas in 12.5 μM [Ca^2+^]_i_, it was ~0.3 mM. This suggests that the conformation of the channel in different [Ca^2+^]_i_ may affect the inner structure of the pore and, consequently, the A9C binding site.

We (this study) and others (Bradley *et al*., 2014; Reyes *et al*., 2015) showed that extracellular A9C only effectively blocked TMEM16A currents at positive *V*_*m*_. This is consistent with a model in which the inhibitory A9C binding site is accessible from the extracellular side and is situated within a region that spans the electric field of the membrane. According to this model, A9C applied from the intracellular side is expected to have no or very little effect on TMEM16A currents. This is because hyperpolarized *V*_*m*_ would not favour entry of intracellular A9C into the channel pore. Indeed, our data show that TMEM16A inhibition by extracellular A9C is enhanced in low [Cl^−^]_ext_, a phenomenon that is consistent with the idea that A9C enters the pore in a competitive manner with permeant anions. However, when applied to the intracellular side of the membrane, A9C produced a small inhibition of the TMEM16A current at positive *V*_*m*_. A possible explanation for these results is that intracellular A9C reached the inhibitory site by crossing the membrane bilayer and then blocking TMEM16A channels from the extracellular side of the membrane. This is plausible considering that A9C contains a hydrophobic region of three aromatic rings, which could enable the drug to cross the membrane lipid bilayer (Qu and Hartzell, 2001; Pusch *et al*., 2002).

The parameter *γ* ≈ 1 derived from fitting [A9C]_ext_‐inhibition relationships with the Hill equation argues that one A9C molecule is sufficient to inhibit one TMEM16A channel. The *δ*_*i*_ value of ~0.6 obtained from the Woodhull analysis suggests that the A9C molecule penetrates approximately 60% into the pore length (voltage field) to reach its inhibitory site. This finding is consistent with reported values of ~60 and ~54% for native *Xenopus laevis* CaCCs (Qu and Hartzell, 2001) and cloned TMEM16A channels (Reyes *et al*., 2015) respectively. Cherian *et al.* (2015) reported a value of 57% for A9C block of cloned TMEM16B channels. This may indicate that TMEM16A and TMEM16B share structural similarity in the pore and may present a similar A9C binding site. Indeed, TMEM16A and TMEM16B have indistinguishable anion selectivity and permeability properties (Adomaviciene *et al*., 2013), which is consistent with shared structural similarity in the permeation pathway. Of note is that the parameter *δ*_*i*_ did not change in the presence of different [Ca^2+^]_i_. This may suggest that the depth of the A9C binding site does not vary in spite of the channel presenting different *P*_*o*_ in various [Ca^2+^]_i_.

Noise analysis revealed that A9C block manifested as an apparent reduction in the channel *P*_*o*_. This is consistent with a mechanism of ‘slow’ block in which the drug remains bound to the channel for a period that extends beyond the duration of the gating cycle of the channel.

### A9C as an allosteric Cl^−^ channel activator

In the presence of a physiological [Ca^2+^]_i_ (i.e. ~300 nM) and in the absence of A9C, pulses to negative *V*_*m*_ elicited instantaneous currents followed by exponential current decays. These exponential current decays reflected the channel's conformational changes leading to channel closure. However, in the presence of A9C and at negative *V*_*m*_, the instantaneous tail currents were described by the sum of two exponential functions. The initial rapid phase presumably represents dissociation of A9C from the inhibiting site, while the slower current decay may represent long lasting A9C‐mediated conformational changes. The relationship between the time‐course of current decay and *V*_*m*_ shifted progressively leftwards as [A9C]_ext_ was increased. This is consistent with the leftward shift in the steady‐state activation curves and the observed changes in channel *P*_*o*_ at a fixed *V*_*m*_ determined via non‐stationary noise analysis.

TMEM16A currents increased by extracellular A9C were not caused by changes in single channel conductance or ion selectivity, nor were they due to alterations in the intrinsic sensitivity of the channel to *V*_*m*_. Thus, it could be inferred that A9C may affect the apparent sensitivity of the channel to Ca^2+^.

### A9C binding site(s) on the TMEM16A channel

The data in Figure 7 indicate that both the extent of current activation and inhibition by extracellular A9C are enhanced in the presence of low Cl^−^. These results are consistent with the idea that Cl^−^ directly competes with A9C binding within the pore and therefore suggest that A9C binds within the anion permeation pathways to exert both inhibiting and activating effects. Furthermore, the electrical distance of the inhibiting and activating sites are indistinguishable (~0.6 in each case), indicating that A9C binds to a region at the same depth of the pore to elicit both inhibition and activation. However, the reduction in extracellular Cl^−^ did not affect the extent of TMEM16A inhibition and activation in precisely the same way. It is well established that tight coupling exists between the permeation pathway and gating in CaCCs as in other Cl^−^ channel types. Thus, it can be envisaged that when extracellular Cl^−^ was lowered, the pore occupancy by Cl^−^ was also reduced. This, in turn, would cause a slight decline in the TMEM16A channel *P*_*o*_ offering more scope for A9C activation. Another possibility is that two separate A9C binding site exist, which are located at an equal depth within the channel pore.

### A9C effect on cloned and native TMEM16A channel

Dual effects (activation and inhibition) of A9C have also been reported for native CaCCs in rabbit pulmonary artery smooth muscle cells (Piper and Greenwood, 2003). For example, native CaCC tail currents at −80 mV increased by ~3‐fold in the presence of 500 μM A9C (Piper and Greenwood, 2003). This effect is comparable with the increase in tail currents we have observed (Figure 1). Furthermore, Piper and Greenwood (2003 showed that A9C did not change the *E*_*rev*_ of native CaCC currents. We also report no change in the *E*_*rev*_ for cloned TMEM16A currents in the absence and presence of A9C. Thus, the biphasic response of TMEM16A‐mediated CaCC currents to A9C may be used as a signature sequence to identify TMEM16A‐mediated currents in a variety of native cells.

### TMEM16A: pathophysiology and potential for therapeutic exploitation

Selective modulators of TMEM16A activity could be of therapeutic use in a number of pathological conditions. For example, TMEM16A is abundantly expressed in vascular smooth muscle (Davis *et al*., 2010; Manoury *et al*., 2010; Thomas‐Gatewood *et al*., 2011; Heinze *et al*., 2014; Wang *et al*., 2015) where TMEM16A channel openers and blockers could be employed to treat hypotension and hypertension respectively. Overexpression of TMEM16A has been reported in pulmonary arteries during pulmonary hypertension (Sun *et al*., 2012), and up‐regulation of Cl^−^ currents has been implicated in the proliferation of pulmonary artery smooth muscle cells (Liang *et al*., 2009). Thus, TMEM16A blockers could be beneficial in pulmonary hypertension by inducing smooth muscle relaxation and possibly by reducing cell proliferation. Small molecules that activate the TMEM16A channel could be used to treat cystic fibrosis. Along with CFTR (Chiaw *et al*., 2011; Donaldson and Galietta, 2013), TMEM16A channels have been identified on the apical membrane of airway epithelia (Ousingsawat *et al*., 2009; Rock *et al*., 2009). Thus, TMEM16A activators may promote Cl^−^ fluxes in these cells to help overcome impaired Cl^−^ transport caused by defective CFTR channels. TMEM16A blockers may also have implications in cancer therapy. Overexpression of TMEM16A promotes tumour cell proliferation in head and neck squamous cell carcinoma, while pharmacological inhibition of TMEM16A current with T16A_inh_‐A01 reduced tumour growth (Duvvuri *et al*., 2012).

### Towards the identification of the A9C binding site(s)

Structural determinants involved in A9C binding have been identified for ClC‐1 channels (Estevez *et al*., 2003). Arginine 646 (R646) and arginine 761 (R761) in *X*. *laevis* TMEM16A have been proposed as potential A9C interactions sites (Reyes *et al*., 2015). Substituting these residues for glutamate reduced the IC_50_ for A9C inhibition measured at various positive *V*_*m*_ (Reyes *et al*., 2015). However, it cannot be excluded that these residues might alter A9C binding/efficacy indirectly. The equivalent residues in the recently derived *Nectria haematococca* TMEM16 X‐ray structure (Brunner *et al*., 2014) appear to be located in a region close to the extracellular environment (R646) or embedded in the protein environment in the vicinity of the Ca^2+^ binding site (R761). Thus, these residues may not directly face the inner portions of the anion permeation pathway in TMEM16A.

The complete identification of TMEM16A residues involved in A9C interaction will likely become possible when the crystal structure of this protein becomes available and the precise structural elements that form the pore are fully elucidated. Homology modelling of TMEM16A based on the *N*. *haematococca* TMEM16 X‐ray structure (Brunner *et al*., 2014) and computational docking of A9C might also offer insights into the location of the A9C binding sites in TMEM16A channels. Elucidating the precise A9C binding sites may aid the design of specific and potent modulators of TMEM16A channel activity which could have both scientific and clinical implications as research tools and medical therapies respectively.

## Conflict of interest

None.

## Author contributions

P. T. designed the research study. C. M. T, A. A. and P. T. designed the experiments. All authors performed the research. C. M. T, A. A., H. G. and P. T. analysed the data. P. T. composed the manuscript, while all authors reviewed and approved the final version of the manuscript.

## Supporting information


**Figure S1** The effect of A9C on the reversal potential of TMEM16A‐mediated whole‐cell currents A) Mean instantaneous whole‐cell current density *versus* *V*_*m*_ relationship obtained from the experiments of Figure 1 conducted in the absence of A9C (control) B) Mean instantaneous whole‐cell current density *versus* *V*_*m*_ relationship obtained from the experiments of Figure 1 conducted in the presence of 3000 µM [A9C]_ext_. The number of experiments was 13 in each case.
**Figure S2** Relationship between τ_B_^‐1^ and [A9C]_ext_ measured at various *V*_*m*_, as indicated. The continuous lines are the best fit of the data with Suppl. Eqn.5.
**Table S1** Parameters (kon and koff) obtained from the fit of the relationship between τ_B_^‐1^ and [A9C]_ext_ measured at various Vm with Suppl. Eqn.5. Ki was calculated as k_off_/k_on_.

Supporting info item
