# Supplementary material for: Mechanism of allosteric activation of TMEM16A/ANO1 channels by a commonly used chloride channel blocker
Source: Br J Pharmacol. 2016 Jan 18;173(3):511–28. doi: 10.1111/bph.13381 (PMC4728427; doi:10.1111/bph.13381)
Supplement: Supplementary file 1 — Supporting info item [file BPH-173-511-s001.pdf]

Ta C.M., Adomaviciene A., Rorsman N., Garnett H. and Tammaro P.

SUPPLEMENTARY INFORMATION

ADDITIONS TO THE METHODS

Electrophysiology

TMEM16A currents were measured with the whole-cell or inside-out configuration of the patch-clamp technique using an Axon 200B amplifier (Molecular Devices, USA) controlled with GE-pulse software (http://users.ge.ibf.cnr.it/pusch/programs-mik.htm). Currents were filtered at 2-5 kHz and sampled at 10 kHz, unless stated otherwise. Pipettes were prepared from borosilicate glass capillary tubes (Harvard Apparatus, USA) using a Narishige PC-10 pipette puller (Narishige, Japan). Pipette tip diameter yielded a resistance of 2.2 to 2.8 MΩ in the working solutions. The bath was grounded through a 3 M KCl agar bridge connected to a Ag-AgCl reference electrode. In whole-cell recordings, the series resistance was usually compensated by ≥70 % to achieve a maximal effective series resistance generally lower than ~5 MΩ. To allow for equilibration of the pipette solution with the cell interior, recordings started 5 min after establishing the whole-cell configuration (Manoury, Tamuleviciute & Tammaro, 2010). Experiments were conducted at 20-22 °C. The cell capacitance was assessed by measuring the area beneath a capacitive transient elicited by a 10 mV step or via the cell capacity compensation circuit of the amplifier.

ADDITIONS TO THE RESULTS

Estimation of K_i at various V_m using the Langmuir formalism

Detailed kinetic analysis of TMEM16A channel activity is likely to require complex models to account for the effects of Ca^{2+} and voltage on channel gating (Hartzell, Putzier & Arreola, 2005). However, a simplified two-state kinetic scheme may be applicable in conditions in which high $[Ca^{2+}]_i$ is maintained to maximally activate the TMEM16A channel. Under these conditions, and assuming that a molecule of A9C binding into the pore of a TMEM16A channel results in the complete block of Cl^- fluxes, the Langmuir formalism applies:

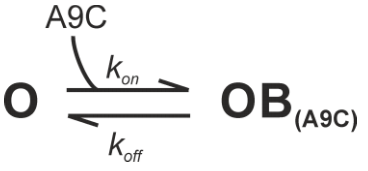

(Scheme I)

Where O represents the conductive state and $OB_{(A9C)}$ is the A9C-blocked state. k_{on} and k_{off} are the association and dissociation constant, respectively and $K_i = k_{off}/k_{on}$ is the apparent equilibrium dissociation constant.

The scheme (I) refers to the following differential equations:

$$\frac{dOB_{(A9C)}}{dt} = [A9C]k_{on}O - k_{off}OB_{(A9C)} \qquad \text{(Suppl. eqn.1)}$$

$$\frac{dO}{dt} = -[A9C]k_{on}O + k_{off}OB_{(A9C)} \qquad \text{(Suppl. eqn.2)}$$

Where O and $OB_{(A9C)}$ describe the probability of being open (unliganded) or blocked, respectively.

Thus, in the presence of a fixed $[A9C]_{ext}$, when the V_m is stepped to a defined test-pulse value:

$$O(t) = O_{\infty} + (O(0) - O_{\infty})\exp\left(-\frac{t}{\tau_B}\right) \qquad \text{(Suppl. eqn.3)}$$

Where O_{∞} is the value of $O(t)$ at the steady state and $O(0)$ is the value of $O(t)$ at the beginning of the test pulse while τ_B is the time constant.

It follows that:

$$O_{\infty} = \frac{1}{1 + \frac{[A9C]}{K_i}} \qquad \text{(Suppl. eqn.4)}$$

$$\tau_B^{-1} = k_{off} + [A9C]k_{on} \qquad \text{(Suppl. eqn.5)}$$

The current decay of various test-pulse V_m for the experiments shown in Figure 3 were fitted with supplementary equation 3 and the resulting τ_B^{-1} plotted *versus* $[A9C]_{ext}$ (Suppl. Fig.2). Supplementary equation 5 was used to fit the τ_B^{-1} obtained at a given V_m, with k_{on} and k_{off} as free parameters (Suppl. Table 1). K_i was calculated as k_{off}/k_{on}.

Suppl. Fig.1

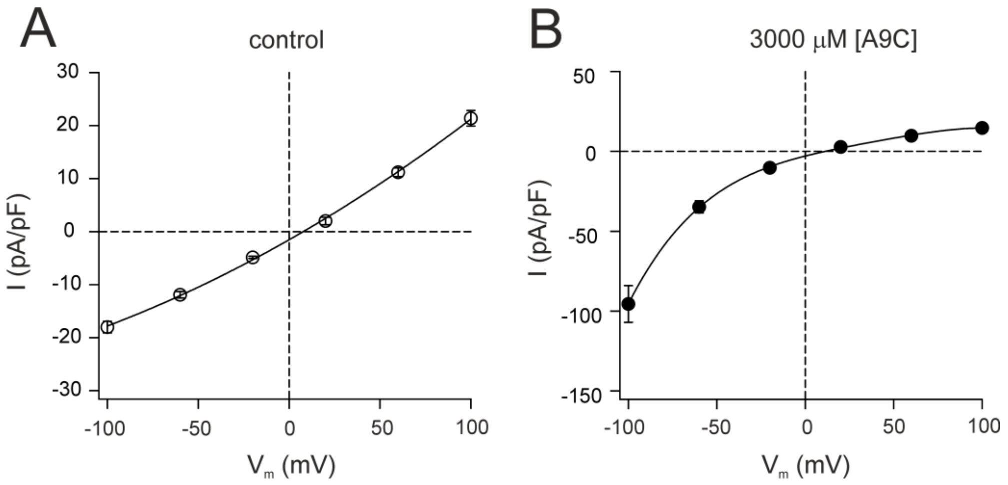

Supplementary Figure 1. The effect of A9C on the reversal potential of TMEM16A-mediated whole-cell currents A) Mean instantaneous whole-cell current density *versus* V_m relationship obtained from the experiments of Figure 1 conducted in the absence of A9C (control) **B)** Mean instantaneous whole-cell current density *versus* V_m relationship obtained from the experiments of Figure 1 conducted in the presence of 3000 µM $[A9C]_{ext}$. The number of experiments was 13 in each case.

Suppl. Fig.2

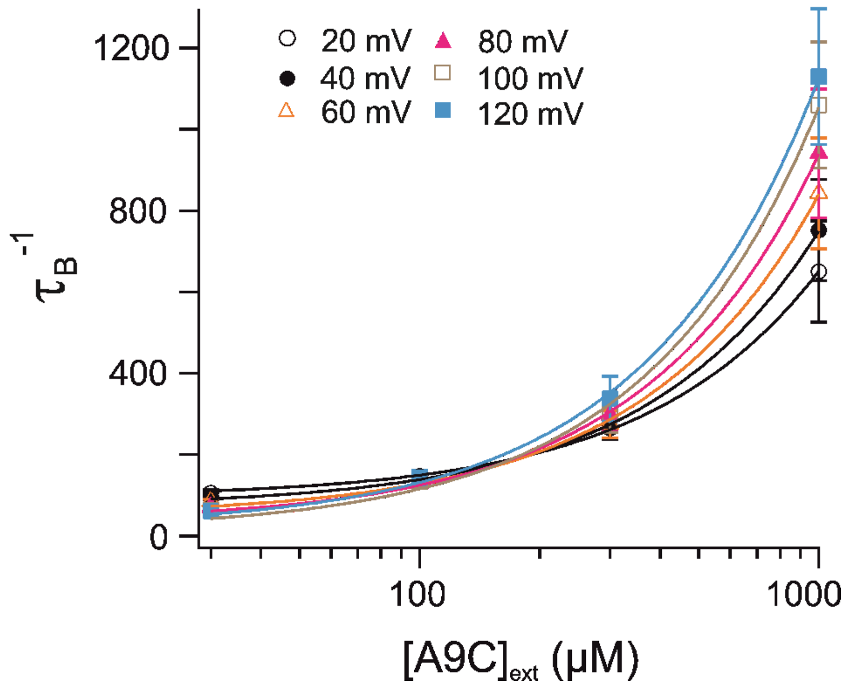

Supplementary Figure 2. Relationship between τ_B^{-1} and $[A9C]_{ext}$ measured at various V_m, as indicated. The continuous lines are the best fit of the data with Suppl. Eqn.5.

SUPPEMENTARY REFERENCE LIST

Hartzell C, Putzier I, & Arreola J (2005). Calcium-activated chloride channels. Annu Rev Physiol 67**:** 719-758.

Manoury B, Tamuleviciute A, & Tammaro P (2010). TMEM16A/Anoctamin 1 protein mediates calcium-activated chloride currents in pulmonary arterial smooth muscle cells. J Physiol 588**:** 2305-2314.

SUPPLEMENTARY TABLES

Supplementary Table 1. *Parameters (k_{on} and k_{off}) obtained from the fit of the relationships between τ_B^{-1} and $[A9C]_{ext}$ with Suppl. Eqn.5. K_i was calculated as k_{off}/k_{on}.*

V_m (mV)	k_{on} ($s^{-1}\mu M^{-1}$)	k_{off} (s^{-1})	K_i (µM)
20	0.56±0.01 (n=8)	94±5 (n=8)	168
40	0.68±0.01 (n=8)	71±9 (n=8)	104
60	0.79±0.02 (n=8)	49±13 (n=8)	62
80	0.90±0.01 (n=8)	34±9 (n=8)	38
100	1.04±0.04 (n=8)	12±25 (n=8)	12
120	1.10±0.03 (n=8)	21±17 (n=8)	19
